# Prospective Prediction of Lapses in Opioid Use Disorder: Protocol for a Personal Sensing Study

**DOI:** 10.2196/29563

**Published:** 2021-12-07

**Authors:** Hannah Moshontz, Alejandra J Colmenares, Gaylen E Fronk, Sarah J Sant'Ana, Kendra Wyant, Susan E Wanta, Adam Maus, David H Gustafson Jr, Dhavan Shah, John J Curtin

**Affiliations:** 1 Department of Psychology University of Wisconsin-Madison Madison, WI United States; 2 Center for Health Enhancement Systems Studies College of Engineering University of Wisconsin-Madison Madison, WI United States; 3 School of Journalism and Mass Communication University of Wisconsin-Madison Madison, WI United States

**Keywords:** digital therapeutics, risk prediction, opioid lapse, mobile phone

## Abstract

**Background:**

Successful long-term recovery from opioid use disorder (OUD) requires continuous lapse risk monitoring and appropriate use and adaptation of recovery-supportive behaviors as lapse risk changes. Available treatments often fail to support long-term recovery by failing to account for the dynamic nature of long-term recovery.

**Objective:**

The aim of this protocol paper is to describe research that aims to develop a highly contextualized lapse risk prediction model that forecasts the ongoing probability of lapse.

**Methods:**

The participants will include 480 US adults in their first year of recovery from OUD. Participants will report lapses and provide data relevant to lapse risk for a year with a digital therapeutic smartphone app through both self-report and passive personal sensing methods (eg, cellular communications and geolocation). The lapse risk prediction model will be developed using contemporary rigorous machine learning methods that optimize prediction in new data.

**Results:**

The National Institute of Drug Abuse funded this project (R01DA047315) on July 18, 2019 with a funding period from August 1, 2019 to June 30, 2024. The University of Wisconsin-Madison Health Sciences Institutional Review Board approved this project on July 9, 2019. Pilot enrollment began on April 16, 2021. Full enrollment began in September 2021.

**Conclusions:**

The model that will be developed in this project could support long-term recovery from OUD—for example, by enabling just-in-time interventions within digital therapeutics.

**International Registered Report Identifier (IRRID):**

DERR1-10.2196/29563

## Introduction

### Background

Opioid use disorder (OUD) is a widespread, intractable disease that devastates the people who suffer from it and their families, friends, and communities. Opioid use is more deadly than other drug use; more than two-thirds of all drug overdose deaths in 2017 and 2018 in the United States involved opioids [1-3]. Improving treatment outcomes for OUD is a critical public health need.

OUD is a chronic, relapsing disease. Many people successfully establish opioid abstinence through pharmaceutical treatments, psychosocial treatments, and peer-support groups [4-7]. These approaches can reduce or eliminate opioid use in the short term but are less effective at supporting successful recovery in the long term [8-10].

Most people with OUD experience setbacks in their recovery. Most people lapse (ie, engage in a single episode of opioid use), and some people who lapse, relapse fully (ie, return to regular, harmful opioid use) [11,12]. Lapses can be triggered by mundane sources such as everyday hassles [13]. Lapses can also occur because maintaining recovery-supportive behaviors is difficult in the long term; over time, people may stop taking medications, engaging with therapy, and attending peer-support groups [14-16]. They may also stop using strategies they learned in treatment and support groups to cope with stress, craving, and other triggers for lapses. These changes in recovery-supportive behaviors over time may increase people’s risk of lapses.

Successful long-term recovery requires continuous lapse risk monitoring and appropriately using and adapting recovery-supportive behaviors as lapse risk changes. Ideally, long-term recovery rests on a foundation of general psychological wellness and involves an awareness of, and defense against, the ever-present risk of lapse [14,17-24]. For example, people in recovery may change their routines and learn new psychosocial habits to prevent and overcome drug cravings (eg, avoiding people and places associated with opioid use and engaging in effortful, deliberate coping when cravings arise). To succeed, they may also return to peer-support groups, re-engage with psychosocial treatment, or restart medications when necessary and if their lapse risk increases.

However, lapses can occur after months or years of seemingly successful recovery, and they may often seem to come without warning [9,25-27]. With improved self-monitoring for lapse risk, people may be better able to adapt their treatments, behaviors, and lifestyle to prevent these lapses. Similarly, if treatment providers were able to accurately monitor the lapse risk of patients in their caseload, they may be able to direct their limited resources toward those patients who are at the greatest risk of lapse.

This protocol paper describes research that aims to build a prospective lapse risk prediction model that can facilitate such improved lapse risk monitoring. Specifically, this lapse risk prediction model will generate temporally precise, ongoing lapse probabilities for people in recovery from OUD. Such a lapse risk prediction model can be situated within a digital therapeutic, that is, a software-based treatment platform that aims to prevent disease or manage disease recovery. Digital therapeutics already support people to manage complex, chronic health issues such as substance use disorders (SUDs) by providing a suite of interventions, information, and interactive tools and services that people can access 24×7 on demand [19,28-36]. Digital therapeutic apps on smartphones are also well-positioned for lapse risk prediction because they can use personal sensing methods to collect low-burden, high-quality information that is necessary for lapse risk prediction [37], deliver lapse risk probabilities directly to people in recovery and their app-connected treatment providers, and provide interventions, information, tools, and services at moments of greatest need (ie, *just in time*) and tailor these supports to the characteristics of the person and their context.

We plan to collect all data necessary to develop a lapse risk prediction model within the Comprehensive Health Enhancement Support System for Addiction (A-CHESS), a digital therapeutic for SUD [19,38,39].

In this paper, we first review previous research on lapse risk prediction, highlighting the importance of understanding lapse risk as resulting from a complex interplay of stable and dynamic risk and protective factors [40]. We then review innovative measurement approaches that make collecting information relevant to lapse risk prediction within digital therapeutics feasible. Next, we describe how machine learning statistical approaches can be used for prospective lapse risk prediction. Finally, we describe the methods we will use to develop this lapse risk prediction model. In the *Conclusions* section, we summarize the potential impact of this research.

### Lapse Risk Prediction

For more than 30 years, research and treatment communities have sought to understand and predict lapses during recovery from SUD [7,33,41-43]. This work has resulted in theoretical accounts of why people lapse and the identification of traits, experiences, and behaviors that confer risk or protection from lapses.

The traits and other stable factors that confer overall lapse risk or protection relate to people’s affective and behavioral tendencies and their history of substance use. People who have a family history of substance use [33], have a long and early personal history of use [44], had severe pretreatment dependence and withdrawal [43], experience more negative emotions than others [33,41,42], struggle with distress tolerance more than others [33,41,42], and have impulsive tendencies [42] are at a greater risk of lapse than others.

However, people’s risk of lapse also fluctuates over time [32,33,41,42,44,45]. Thus, stable individual differences alone are not sufficient to predict lapse [7,45-49]. People’s behavior and experiences and the monthly, weekly, and daily changes and events in their lives affect their moment-to-moment lapse risk.

The dynamic (ie, temporally varying) factors that confer lapse risk or protection include people’s engagement with treatment [50], exposure to use-related cues in their physical and social environments [51], and their wellness, including their stress, cravings, and affective experiences [52-54]. People are at lower risk of lapsing when they attend support groups [55] and take medications as prescribed [56]. People are at higher risk of lapsing when they see people and visit places associated with their past use [51,57], experience job loss [55,56], have more severe pain than usual [51,58], and have more cravings than usual [51-53].

### Personal Sensing for Prospective Lapse Risk Prediction

The research described in this paper focuses on prospective lapse risk prediction for clinical implementation. This requires different measurement approaches from those of previous theory-driven research. Earlier research on the theoretical causes of lapses has focused on testing inferences about these causal factors. Testing causal inferences requires measuring (or better still, manipulating) small numbers of factors. Therefore, this earlier research generally measured or manipulated a select few putative causal factors for lapse once or periodically, depending on how often the factors of interest change (eg, every few months or weeks, daily, or multiple times a day) [59,60]. This research seeks to identify causal factors rather than achieve high predictive accuracy for lapses.

Prospective lapse risk prediction for clinical implementation likely requires measuring many lapse-related factors to account for sufficient variance in lapse outcomes to make accurate predictions. In addition, these factors must be measured frequently enough to capture meaningful variation over time. For example, accurate prediction of the probability of lapse in the next 24 hours may require knowing the status of, and recent changes in, hundreds of factors. Some factors relevant to lapse risk are stable individual differences, but others are dynamic and may change quickly (within hours) or slowly (in weeks or months). Prospective lapse risk prediction requires a measurement strategy that can accommodate continuous, longitudinal measurement of some factors and place minimal burden on people despite capturing information about hundreds of factors.

Self-report methods alone cannot support prospective risk prediction. Self-report is well suited for measuring subjective states, including theoretical causes of lapse, such as affect and pain. However, collecting self-report requires active effort from the individual, which limits the frequency and quantity of factors that self-report can measure.

Recent technological innovations enable measurement approaches that can complement self-report with respect to the need for accurate, prospective risk prediction. Specifically, personal sensing methods leverage sensing technologies in smartphones, wearable devices, social media, and computers to capture information longitudinally about people’s naturalistic environments, behavior, social interactions, thoughts, and affect [28,37]. By definition, personal sensing methods provide naturalistic in situ and longitudinal measurement.

Personal sensing methods can be active or passive. Active methods require people to take actions to provide measurement, including self-report. For example, ecological momentary assessments are brief self-report surveys focused on momentary states. People may complete these surveys multiple times per day to provide in situ longitudinal measurement of their subjective experiences. Other examples of active personal sensing include audio or video check-ins, where people describe a positive event in the past, a negative event in the past, or something they are looking forward to in the future.

In contrast, passive personal sensing methods can measure processes with little burden placed on the individual. For example, software monitoring of smartphone call logs and monitoring of geolocation through smartphone location services are both passive personal sensing methods. In some instances, these passive methods can provide lower burden or even privileged access to measure people’s behavior or subjective experiences. For example, rather than using self-report surveys to collect information about people’s social contacts, exercise, or recent activities, data from people’s smartphones can be used as a proxy for these factors. Smartphone call and text message logs can reveal how much social contact someone has had. Geolocation and accelerometer data can be used to estimate people’s exercise and activities or even detect long periods of social isolation at home.

Both active and passive personal sensing methods are now possible within digital therapeutic smartphone apps such as A-CHESS. Smartphones house sensors and software that can capture information such as geolocation and movement, audio and video recordings, phone use patterns, call and SMS text message logs, and SMS text message content. Digital therapeutics can access smartphone hardware and software to collect and integrate these data. These raw data form the inputs from which to derive predictors of lapse risk.

### Deriving Lapse Risk Predictors

The information that smartphones can collect can produce powerful, theoretically informed predictors of lapse risk. Self-report surveys delivered through smartphones can capture predictors such as people’s substance use history, stable tendencies related to risk, and monthly or daily changes in people’s craving, affect, experience of stressful or pleasant events, and other risk-related subjective experiences. Geolocation data can capture the frequency and duration of visits to places or movement patterns that may indicate lapse risk (eg, excessive time spent in a location and late-night excursions). Phone call and text message logs can capture the number and pattern of communications with friends or family. The content of people’s text messages can indicate their mood, stress, experiences of craving, and other dimensions of their mental health [61]. How often and for how long people use their digital therapeutic app’s features can indicate their motivation, commitment, and engagement in recovery-supportive behaviors.

Passive personal sensing information collected from digital therapeutic smartphone apps can be made even more powerful by gathering additional intrapersonal context to better characterize the raw data, for example, by identifying frequent social contacts and asking people for additional information about them. The frequency, timing, and duration of phone calls can be enhanced with self-reported contextual information about relationship closeness and perceived recovery support provided by these contacts. For example, 3 brief morning phone calls to a close friend may signal increased lapse risk, but 3 brief morning phone calls to an internet service provider likely do not. Similarly, patterns in geolocation data can be enhanced with public or self-reported context about type (eg, hospital, bar, restaurant, or a friend’s residence) and meaning (eg, recovery supportive and typically pleasant or unpleasant) of the places visited. For example, 5 hours spent at a hospital emergency department may signal increased lapse risk, but 5 hours spent at a recovery-supportive friend or family member’s apartment building likely does not.

Critically, contextual information about important people and places can be collected with relatively little burden. Most people have a relatively small, stable set of frequent social contacts and frequently visited places [62-64]. In a previous project, our research group identified a method of collecting this self-reported contextual information. Specifically, contextualizing information for geolocation and cellular communications data was collected in a brief self-report survey administered monthly over a period of 3 months [65]. After a month of personal sensing data collection, frequent contacts (ie, more than 2 interactions per month) and frequently visited places (ie, places visited more than twice a month) were identified. People answered a brief set of questions about each frequent contact (eg, relationship type; perceived closeness; supportiveness of recovery; typical pleasantness or unpleasantness of interactions; and typical support for, or risk to, recovery) and each place (eg, place type, associated activities, typical pleasantness or unpleasantness of visits, and typical supportive or risk-related effect of visits on recovery). This contextual information can be used to enrich the predictive signal of passively sensed cellular communications and geolocation data.

### Modeling Prospective Lapse Risk With Machine Learning

Digital therapeutics can leverage smartphone tools and sensors to feasibly measure and derive risk-related predictors, but accommodating these predictors in a statistical risk prediction model poses a new challenge for prospective lapse risk prediction. Lapse risk is known to relate to a large number of stable and dynamic factors. It is also theorized to result from complex interactive and nonlinear functions of these factors [30,32,33,40,45,66]. Therefore, the statistical models used must support high-dimensional (ie, many predictors) and complex data-generating processes to achieve the high predictive accuracy necessary for clinical implementation. Furthermore, for useful clinical implementation, these statistical models must generalize well when applied to new people and settings and not just those that the model was trained on. Analytic approaches that are typical of theoretical research on lapse risk, such as generalized and multilevel linear models, are not well suited to these challenges. In contrast, machine learning approaches have been developed specifically to achieve these goals [67,68].

High-dimensional sets of predictors pose challenges to many statistical modeling approaches. On the one hand, too many predictors (correlated predictors in particular) may yield overfit, unstable models that vary strongly based on the data used to develop them (ie, high variance), which can compromise model generalizability; on the other hand, too few predictors (as well as other constraints on model characteristics) yield underfit models that may consistently over- or underestimate an outcome (ie, high bias). Machine learning uses various techniques (eg, regularization and hyperparameter tuning) to optimize these bias-variance trade-offs to accommodate high-dimensional sets of predictors while reducing overfitting to the data used for model development. This allows machine learning models to take advantage of high-dimensional predictor spaces to capture complex relationships and patterns learned from these data.

Machine learning also provides rigorous methods to develop and evaluate models in separate data [67]. Cross-validation techniques can be used with a subset of data (ie, the training set) to identify the best-performing model. This best-performing model is selected by cross-validation to maximize its ability to be generalized to new people. This model’s performance can then be explicitly evaluated in previously held-out data (ie, the test set) that were not used for model development or selection. This cross-validation procedure allows for more realistic estimates of the performance of the model when it is generalized for use with new people.

### Study Objective and Overview

The objective of this study is to develop a highly contextualized lapse risk prediction model that forecasts the ongoing probability of lapse among adults in recovery from OUD. This prediction model will be developed using predictors derived from raw data collected by active and passive personal sensing methods within a digital therapeutic smartphone app, A-CHESS. We will enroll people in their first year of recovery and follow them longitudinally for 1 year. We will recruit a sample that is diverse in their recovery stability, race, ethnicity, and geographic setting (urban, suburban, and rural residence) to provide the raw data necessary to develop a prospective risk prediction model that generalizes well. We will use contemporary machine learning methods to train this prospective risk prediction model and evaluate its performance with new (not previously seen during training) people.

## Methods

### Participants

We will enroll 480 adults receiving medication-assisted treatment for OUD. We are recruiting these participants using targeted national digital advertising and collaborations with treatment providers at medication-assisted treatment clinics. Our recruitment strategy has been designed to create a diverse sample with respect to recovery stability, demographics (sex, age, race, and ethnicity), and geographic setting (urban, suburban, and rural residence). We do not exclude participants for comorbid SUD or other psychopathologic conditions. To enroll, participants must be aged ≥18 years, fluent English speakers, stable recipients of medication-assisted treatment (defined as taking monthly medication regularly or daily medication on most days or every day) for at least 1 month but no longer than 12 months, and Android smartphone users with an active cellular plan.

We compensate participants for completing brief phone meetings with study staff for initial enrollment and training. Participants earn US $20 per hour for the time they spend in these phone meetings and US $20 for completing training materials. We also compensate participants for completing study tasks, and we award bonuses to participants when they exceed the minimum compliance requirements for study tasks. Participants earn a nominal amount for each daily survey and daily video check-in and are awarded bonuses for completing at least 24 of these per month. Earnings amount to a maximum of US $15 each month for completing daily surveys, US $10 each month for submitting daily video check-ins, US $10 for completing the intake or monthly survey, and US $15 for keeping data sharing (eg, location services and cellular communications) enabled the entire month. In addition to paying participants for completing tasks, we pay US $50 per month to participants’ cell phone providers to offset the costs of maintaining a phone plan.

### Procedure

Participants are recruited through partnerships with health care systems across the United States and through digital advertising (eg, Facebook advertisements and posts to opioid recovery–relevant subreddits on Reddit). Participants are screened by staff or by completing a brief web-based survey. Interested and eligible participants speak with project staff on the phone to learn more about the study and provide informed consent. Consenting participants provide demographic information, install the app, and complete web-based training.

After enrollment, participants will provide information about themselves and their lapses for a year, information about stable risk-related factors in an intake survey, and information about dynamic risk-related factors through different means. Participants will provide continuous data relevant to some dynamic factors through passive personal sensing of their cellular communications, geolocation, and use of A-CHESS. Every month, participants will actively provide information about dynamic factors through a survey (eg, changes to their housing and employment and information about their mental and physical health and health care). Participants will also provide contextual information about important people with whom they communicate and the places they visit. Every day, participants will provide information about dynamic factors such as their affect, pain, cravings, and motivation by recording a brief (15-30 seconds) selfie-style video check-in and in a brief self-report survey. In their daily self-report survey, participants will also provide information about their lapses (ie, uses of opioids for nonmedical reasons), indicating when they happened by selecting among 6-hour intervals that span their study enrollment. All study data will be collected through A-CHESS.

During the first week of enrollment, study staff will meet with participants by phone to answer questions they have about the study and app and to help them troubleshoot technical issues. Additional meetings with study staff are arranged as needed to resolve technical issues. Training and support materials (eg, infographics and video guides) remain available to participants through A-CHESS. When participants complete the study, discontinue, or withdraw, they will have a brief debrief phone call with study staff.

### A-CHESS Digital Therapeutic App

A-CHESS is the digital therapeutic smartphone app that we use in the study. A-CHESS houses a suite of resources and tools for people in recovery from SUD [19,38,39]. The features that A-CHESS offers were designed with guidance from the Marlatt cognitive behavioral model of relapse prevention (Marlatt and George [69]) and self-determination theory (Ryan and Deci [70]). The app aims to reduce relapse risk through features such as a discussion board, guidance on coping with cravings, a library of resources, a gratitude journal, and alerts if a user is near a self-identified high-risk location (see the URL [71] for a detailed description of the app’s features).

As of July 2021, A-CHESS has been used by more than 7000 people and 60 treatment centers nationwide. A-CHESS has been developed and refined using techniques from a user-centered design where feedback from key users of the system is sought early and often. User stories and scenarios, participatory design sessions, one-on-one contextual interviews, usability testing, and focus groups have designed and evaluated each of the recovery tools.

A-CHESS provides recovery support to participants enrolled in the study and also collects information relevant to lapse risk using personal sensing methods. A-CHESS administers the self-report surveys and collects passive personal sensing measures such as geolocation and cellular communications (ie, text message content and logs and phone call logs). In addition, the digital therapeutic app has features that support user privacy, such as password protection and adjustable settings for data sharing.

### Measures

#### Overview

Detailed descriptions of the measure items, sources, and administration (eg, instructions) are available on the website [71]. We use these measures to derive predictive features associated with stable individual differences and temporally dynamic risk factors. The granularity of the dynamic risk factors varies across measures from monthly to daily to moment by moment as described below. We collect all measures through personal sensing using A-CHESS on participants’ smartphones.

#### Monthly Survey

The monthly survey includes measures of stable individual differences and dynamic risk factors. There is some variation in the specific measures that are included in different months as described below. However, all monthly surveys take less than 30 minutes to complete on average.

The first monthly survey is an intake survey that is administered shortly after participants enroll in the study. This survey includes measures related to stable individual differences. Specifically, it measures demographics, lifetime substance use history (items adapted from the World Health Organization Alcohol, Smoking and Substance Involvement Screening Test version 3.0 [72]); opioid treatment history; Diagnostic and Statistical Manual of Mental Disorders, Fifth Edition, OUD diagnostic criteria for the year before starting medication-assisted treatment [73]; distress tolerance (items selected from the Distress Tolerance Scale [74]); pain catastrophizing (items adapted from the Pain Catastrophizing Scale and Pain Catastrophizing Scale for Children [75,76]); personality traits relevant to psychopathology (Personality Interview Guide from Diagnostic and Statistical Manual of Mental Disorders, Fourth Edition, Brief Form [77,78]); adverse childhood experiences (items selected from the Adverse Childhood Experiences Questionnaire [79]); and trauma experience [80].

The first and later monthly surveys also include measures related to dynamic risk factors. This includes measures of life circumstances (eg, employment status and living situation); social connectedness (adapted from the Medical Outcomes Study Social Support Survey [81]); romantic relationship quality (items selected from the Relationship Assessment Scale [82]); psychiatric symptoms (items selected from the Behavior and Symptom Identification Scale-32 [83]); pain and anticipated pain treatment (items adapted from the Wisconsin Brief Pain Questionnaire and the Pain, Enjoyment, General Activity Scale [84,85]); stress (items selected from the Perceived Stress Scale [86]); quality of life (items adapted from the World Health Organization Quality of Life Assessment [87]); substance use (adapted from the World Health Organization Alcohol, Smoking and Substance Involvement Screening Test version 3.0 [72]); opioid use; opioid recovery satisfaction and motivation; other recovery goals; and questions about treatment use, adherence, and perceived efficacy, including questions about medication-assisted treatment, self-help meetings, counseling, psychiatric medication, and detox or other inpatient residential treatment [88,89].

The later monthly surveys also contain questions about the intrapersonal and subjective context associated with people and places with which the participant has frequent contact or visits. The monthly surveys administered at 6 months and the final month of the study also include questions about participant perceptions of the acceptability and burden associated with each of the major categories of personal sensing signals (eg, daily survey, video check-ins, and passive monitoring of geolocation).

#### Daily Survey

The daily survey includes measures of dynamic risk factors that are collected with greater temporal granularity than in the monthly survey. It becomes available at 5 AM CST and can be completed at any time over the next 24 hours. This survey is brief (16 items) and takes approximately 1 minute to complete.

This survey collects specific reports of the date and time of any recent opioid use for nonmedical reasons that participants have not already reported. These reports serve as the primary outcome for the lapse risk prediction model. Participants also report any other drugs that they have used in the past 24 hours by selecting all that apply from a list of substance categories with examples (eg, alcohol and stimulants). The daily survey includes items related to mood, pain, sleep quality, urges to use opioids, risky situations, stressful and pleasant events, and use of medications associated with their treatment in the last 24 hours. The daily survey concludes with items related to participants’ motivation and confidence to continue to avoid using opioids for nonmedical reasons over the next week.

#### Daily Video Check-In

Each day, participants record a short video check-in using their front-facing phone camera. This video captures their facial expressions and voice as they reflect on recent or expected pleasant or unpleasant events or experiences in the near future. The daily video check-in becomes available at 5 AM CST each day and can be completed at any time over the next 24 hours. It takes less than 1 minute to complete.

#### Moment-by-Moment Geolocation

We use participants’ smartphone location services (accessed through A-CHESS) to passively collect their moment-by-moment geolocation (ie, latitude and longitude). Participants’ time-stamped geolocations are updated automatically every 1.5-15 minutes, depending on their movement.

As described previously, we increase the predictive strength of geolocation data by augmenting it with self-reported subjective contexts. Therefore, the monthly survey includes questions about the places that participants frequently visit (ie, 2 or more times per month). We detect these frequently visited places automatically each month through algorithms that review the previous month’s geolocation data. For each frequently visited place, participants describe the type of place, what they typically do there, the general frequency of pleasant and unpleasant experiences associated with the place, and the extent to which spending time there supports or undermines their recovery. When available, public information (eg, through OpenStreetMap) about these places will also be used to contextualize these data.

#### Cellular Communications

Participants’ cellular communications are passively collected using A-CHESS. This includes the timestamps of all phone calls and SMS text messages, whether calls and SMS text messages are incoming or outgoing, the phone number of the other party, and the name of the contact if it is saved in participants’ phones. For phone calls, the duration of the call is recorded. For SMS text messages, the complete SMS text message content is recorded, excluding any sent or received images.

We potentially increase the predictive strength of the information collected about cellular communications by augmenting it within a subjective intrapersonal context. The monthly survey includes questions about the people with whom the participant has frequent contact (eg, more than 2 calls or 4 SMS text messages per month). We detect these frequent contacts automatically each month through algorithms that review the previous month’s cellular communications. For each frequent contact, participants describe the nature of their relationship with the contact, the general frequency of pleasant and unpleasant interactions associated with the person, and the extent to which interactions with the contact support or undermine their recovery.

#### Digital Therapeutic Use

Participants’ overall use of A-CHESS, including engagement with specific recovery support features, will be collected in time-stamped logs. A-CHESS also captures the comments that participants post about recovery-related media, their activity on A-CHESS discussion boards, and the messages they send within the app.

### Statistical Analyses

#### Machine Learning Overview

Machine learning is a subfield of computer science that offers an alternative analytic approach ideally suited for both precise prediction and generalizability [67,68]. Machine learning models can consider high-dimensional sets of predictor variables (ie, features) simultaneously. Using stable and dynamic data sources, we can engineer thousands of features to be used for prediction (eg, individual risk signals, interactions among stable and dynamic risk signals, and intrapersonal change in scores and responses over time). Machine learning models can take advantage of this high-dimensional feature space to capture complex relationships and patterns learned from the data. However, there is still some cost in including a very large number of features.

In addition, machine learning provides rigorous methods to develop and evaluate models in separate data [67]. Consequently, models generalize well to new data because they are evaluated on out-of-sample prediction. We will use cross-validation techniques with training data to select among a variety of model configurations that differ with respect to the statistical algorithm (and associated hyperparameter values) and feature sets. This approach will allow us to consider models that allow us to restrict ourselves to only passive (ie, low burden) features or remove features with high rates of missing data (as an alternative definition of burden and tolerability). Therefore, we will be able to examine both predictive accuracy and implementation-relevant considerations such as participant burden. We will estimate final model performance in held-out test data.

#### Feature Engineering and Preprocessing

We will use features (ie, predictors) derived from actively and passively collected personal sensing data to build temporally precise machine learning prediction models for lapse risk for different time intervals (eg, daily and weekly). We will follow general recommended practices for data preprocessing and feature processing in machine learning [67,68]. Although procedures differ to some degree based on the specific candidate machine learning algorithm generally, we will review descriptive statistics for data coding errors, apply power transformations to highly skewed features used in linear machine learning models, center and scale all features (unit variances), and dummy code categorical variables. We will remove features with near-zero variance using standard computations implemented in the tidymodels packages in R [90]. For high-dimensional data sources (ie, natural language), we will evaluate a variety of feature extraction methods that reduce dimensionality (eg, Linguistic Inquiry and Word Count [91], singular value decomposition [92], and Word2Vec [93]). All our candidate machine learning algorithms are tolerant of missing data for events. Specifically, missing data imputation procedures can be applied at the level of the feature representation functions [94,95].

#### Candidate Machine Learning Algorithms

We will evaluate these features within a small set of candidate contemporary machine learning (statistical) algorithms. These include Random Forest [96,97], Penalized Logistic Regression (Lasso, Sparse Group Lasso, and Elastic Net) [67,97-101], Multilayer Perceptron Neural Networks [102], and Support Vector Machines [103,104].

These algorithms were intentionally selected to be complementary with respect to several key features that may affect their relative performance (eg, parametric vs nonparametric models and linear vs nonlinear models). They also vary with respect to their flexibility, model complexity, and sample size requirements such that they will likely differ in their ability to address bias-variance trade-offs in the prediction of new data, depending on the true population model underlying the observed data [67,68]. Finally, all these algorithms are well established with documented good *out of the box* performance [67,68].

These algorithms vary with respect to the degree of feature selection performed automatically during training. Critically, Lasso and Sparse Group Lasso will yield sparse solutions at the level of individual features and groups of features organized around data sources (eg, moment-by-moment location, cellular communications, and daily survey). If these sparse solutions perform well, they may be preferred because the final model will need fewer data sources with associated easier implementation and lower user burden. We will also manually evaluate model performance with reduced feature sets (eg, dropping daily surveys) for algorithms that do not handle this automatically during training (eg, Random Forest).

#### Model Training and Evaluation

Model training, hyperparameter tuning, and best model selection will be accomplished in a subset of the data (ie, training set) using repeated grouped k-fold cross-validation. We plan to use a variety of techniques (eg, resampling techniques such as upsampling and Synthetic Minority Oversampling Technique and weighted penalties for minority class) within the training set to accommodate the unbalanced nature of the outcome (lapses are expected to be infrequent). We plan to hold out an independent test set that will not be used for model training or selection. The final performance of the best model configuration will be evaluated on independent data (ie, test set) that were not included in the training set. We will characterize the performance of this best model using standard metrics that are appropriate for unbalanced data (eg, balanced accuracy and area under the receiver operating characteristic curve).

## Results

The National Institute on Drug Abuse funded this project (R01DA047315) on July 18, 2019, with a funding period from August 8, 2019, to June 30, 2024. The Institutional Research Board of the University of Wisconsin-Madison Health Sciences approved this project on July 9, 2019. We began enrolling pilot participants on April 16, 2021. These pilot participants met the inclusion criteria and are being used to test all procedures, personal sensing methods, and the implementation of A-CHESS. Full enrollment began in September 2021. We plan to recruit participants for approximately two-and-a-half years.

## Discussion

### Principal Findings

OUD is a widespread condition characterized by lapses and relapses that can threaten people’s lives and well-being even years into recovery. People often fail to anticipate lapses and relapses, resulting in failure to seek support or use effective preventive strategies when they are at risk of lapse. Smartphone technology enables people to access continuing care for recovery through digital therapeutics. Integrating real-time lapse risk prediction within these digital therapeutics has the potential to support sustained recovery by offering treatment or intervention resources and services to people before a lapse or relapse occurs (eg, just-in-time interventions).

This paper describes the rationale and method of an ongoing, grant-supported project to develop a highly contextualized lapse risk prediction model for people in recovery from OUD. Completing the project will involve collecting information about risk-related factors and lapses from an estimated 480 American adults in recovery from OUD. Information will be collected using a digital therapeutic smartphone app, using both self-report and passive personal sensing methods. The model this project will develop could be used as part of a risk prediction system that would support long-term recovery from OUD, for example, by enabling just-in-time interventions within digital therapeutics.

Bridging the gap between a risk prediction model and a functional risk prediction system that is integrated into a usable digital therapeutic is complex and well beyond the scope of a single R01-supported project. Implementing risk prediction in a way that could prevent lapses requires better, contextualized understanding of the biases in the risk prediction model, effective messaging, costs and benefits of sharing risk predictions with users and treatment providers, and the costs and benefits of different types of information for prediction. Without careful research focused on the details of implementation and without the full understanding and consent of the users, the lapse risk prediction system this line of research aims to produce could cause more harm than good. A system that uses a predictive algorithm to calculate risk from sensitive measures such as cellular communications and geolocations and then communicates that risk to third parties could function as a surveillance system rather than support tool. Furthermore, such a system could perpetuate inequities in recovery (eg, if the algorithm systematically over- or underpredicted lapse risk for people from historically marginalized groups).

### Conclusions

To advance collective understanding of these issues and to help inform future research, our project will provide insights about the feasibility, costs, and benefits of different risk prediction systems. For example, our analytic approach involves training sparse models that predict using less information than is available. In addition, our analysis approach will allow us to assess the performance of the models that rely on information that we know or observe to be less burdensome to participants based on both self-reported burden and behavioral compliance.

This project will complete an essential step toward a critical public health goal: improving outcomes among people with OUD. Effective treatments for OUD exist, but the treatments and behaviors required for achieving long-term recovery are challenging to maintain. Knowing when lapses are likely to occur can provide people with information and motivation to engage in recovery-supportive activities and can help treatment providers care for their patients.

## Appendix

Multimedia Appendix 1 Peer-reviewer report from the Addiction Risks and Mechanisms Study Section - Risk, Prevention and Health Behavior Integrated Review Group (National Institutes of Health).

## Abbreviations

A-CHESS: Comprehensive Health Enhancement Support System for Addiction
OUD: opioid use disorder
SUD: substance use disorder
