# Supplementary material for: Prospective Prediction of Lapses in Opioid Use Disorder: Protocol for a Personal Sensing Study
Source: JMIR Res Protoc. 2021 Dec 7;10(12):e29563. doi: 10.2196/29563 (PMC8693201; doi:10.2196/29563)
Supplement: Multimedia Appendix 1 [file resprot_v10i12e29563_app1.pdf]

SUMMARY STATEMENT
(Privileged Communication)

PROGRAM CONTACT:
Shelley Su
301-402-3869
shelley.su@nih.gov

Release Date: **03/05/2019**
Revised Date:

Application Number: **1 R01 DA047315-01A1**

Principal Investigators (Listed Alphabetically):

CURTIN, JOHN J. (Contact)
SHAH, DHAVAN

Applicant Organization: UNIVERSITY OF WISCONSIN-MADISON

Review Group: **ARM**
Addiction Risks and Mechanisms Study Section

Meeting Date: **02/11/2019**
Council: **MAY 2019**
Requested Start: **08/01/2019**
RFA/PA: **PA18-484**
PCC: **CM/ZIS**

Project Title: **Contextualized daily prediction of lapse risk in opioid use disorder by digital phenotyping**
SRG Action: **Impact Score:25 Percentile:8**
Next Steps: **Visit https://grants.nih.gov/grants/next_steps.htm**
Human Subjects: 30-Human subjects involved - Certified, no SRG concerns
Animal Subjects: 10-No live vertebrate animals involved for competing appl.
Gender: 1A-Both genders, scientifically acceptable
Minority: 1A-Minorities and non-minorities, scientifically acceptable
Children: 3A-No children included, scientifically acceptable

Project Year	Direct Costs Requested	Estimated Total Cost
1	498,430	760,764
2	496,802	758,279
3	496,802	758,279
4	496,802	758,279
5	498,430	760,764
TOTAL	2,487,266	3,796,363

ADMINISTRATIVE BUDGET NOTE: The budget shown is the requested budget and has not been adjusted to reflect any recommendations made by reviewers. If an award is planned, the costs will be calculated by Institute grants management staff based on the recommendations outlined below in the COMMITTEE BUDGET RECOMMENDATIONS section.

1R01DA047315-01A1 Curtin, John

RESUME AND SUMMARY OF DISCUSSION: This application proposes to develop, train, validate, and test lapse prediction models for opioids, alcohol, and other drugs in a sample of abstinent opioid users, combining static relapse risk signals with a comprehensive range of time-varying risk signals assessed via smartphone during a 12-month observation period. The resubmission is responsive to the prior review and reviewers remained enthusiastic about the study's very high public health significance. The application also retains its many original strengths including the exceptional research team (now improved with additional addiction expertise), and the innovative and exciting constellation of data streams used to derive the predictive algorithms (e.g., self-report surveys, ecological momentary assessment, video check-ins, text message and voice calls logs, text message content, GPS locations, physical activity). The resubmission is improved with a more comprehensive assessment battery (e.g., pain, physical health, medication-assisted treatment) and additional methodological and analytic details. New pilot data partially assuage concerns about feasibility, though Aim 3's implementation plan remains somewhat underdeveloped. Overall the strengths of this significant and innovative application easily outweigh its weaknesses and the committee concluded that the study's potential impact is high.

DESCRIPTION (provided by applicant): Opioid use disorder is increasingly widespread, leading to devastating consequences and costs for patients and their families, friends, and communities. Available treatments for opioid and other substance use disorders (SUD) are not successful at sustaining sobriety. The vast majority of people with SUD relapse within a year. Critically, they often fail to detect dynamic, day-by-day changes in their risk for relapse and do not adequately employ skills they developed or take advantage of support available through continuing care. The broad goals of this project are to develop and deliver a highly contextualized, lapse risk prediction models for forecasting day-by-day probability of opioid and other drug use lapse among people pursuing drug abstinence. This lapse risk prediction model will be delivered within the Addiction-Comprehensive Health Enhancement Support System (A-CHESS) mobile app, which has been established by RCT as a state-of-the-art mHealth system for providing continuing care services for alcohol and substance use disorders. To accomplish these broad goals, a diverse sample of 480 participants with opioid use disorder who are pursing abstinence will be recruited. These participants will be followed for 12 months of their recovery, with observations occurring as early as one week post-abstinence and as late as 18 months post-abstinence across participants in the sample. Well-established distal, static relapse risk signals (e.g., addiction severity, comorbid psychopathology) will be measured on intake. A range of more proximal, time-varying opioid (and other drug use) lapse risk signals will also be collected via participants' smartphones. These signals include self-report surveys every two months, daily ecological momentary assessments, daily video recovery "check-ins", voice phone call and text message logs, text message content, moment-by-moment location (via smartphone GPS and location services), physical activity (via smartphone sensors), and usage of the mobile A-CHESS Recovery Support app. The predictive power of these risk signals will be further increased by anchoring them within an inter-personal context of known people, locations, dates, and times that support or detract from participants' abstinence efforts. Machine learning methods will be used to train, validate, and test opioid (and other drug) lapse risk prediction models based on these contextualized static and dynamic risk signals. These lapse risk prediction models will provide participant specific, day-by-day probabilistic forecast of a lapse to opioid (or other drug) use among opioid abstinent individuals. These lapse risk prediction models will be formally added to the A-CHESS continuing care mobile app at the completion of the project for use in clinical care. These project goals position A-CHESS to make relapse prevention and recovery support, information, and risk monitoring available to patients continuously. Compared to conventional continuing care, A-CHESS will provide personalized care and be available and implemented during moments of greatest need. Integrated real-time risk prediction holds substantial promise to encourage sustained recovery through adaptive use of these continuing care services.

PUBLIC HEALTH RELEVANCE: The project's goals are to develop and deliver a real-time model for forecasting day-by-day opioid use lapse among abstinent patients with opioid use disorder. This lapse prediction model will be integrated into an existing, validated mHealth app to encourage sustained recovery through adaptive use of continuing care services.

CRITIQUE 1

Significance: 1
Investigator(s): 1
Innovation: 1
Approach: 3
Environment: 1

Overall Impact: This revised R01 application from an established investigative team proposes to develop an opioid (aim 1) and other drug (aim 2) lapse risk prediction model via a combination of active and passive monitoring of a variety of static and dynamic lapse risk factors implemented via the Center for Health Enhancement Systems Studies – Addiction (A-CHESS) mobile health (mHealth) platform. The application is significant and innovative for using multiple data sources to attempt to predict opioid and other drug lapses over the course of one year following an initial recovery attempt. Individuals (n=480) with opioid use disorder will be recruited locally and nationally (via Facebook) and be assessed using daily ecological momentary assessment surveys, daily video check-ins, bimonthly surveys, and continuous (passive) monitoring of GPS data, A-CHESS recovery support, and text mining of voice calls and text messages. Preliminary data from the highly qualified research team in an excellent environment provides evidence of feasibility. The investigators were mostly responsive to prior critiques; however minor weaknesses remain. Aim #3, to implement the risk prediction tool in A-CHESS, remains underdeveloped and it is unclear how the tool will be implemented and for whom. Barriers to implementation and whether the tool will be implemented if it is not highly predictive are not considered or discussed. Second, the application gives very little attention to sex as a biological variable. Despite these minor weaknesses the application has many strengths and the proposed research is likely to have high overall impact.

1. Significance:

Strengths

- Opioid and other drug relapse in the first year following a treatment attempt is a significant public health problem. Theoretical models of relapse suggest a dynamic process, yet most studies of relapse have relied primarily on static, self-report measurement, which has limited predictive utility. Developing a tool that could proactively assess risk in daily life would be highly significant in addressing the opioid epidemic and difficulties in treating other substance use disorders.
- The investigative team has provided preliminary data in support of the scientific premise for the proposed work, which suggests they have been successful in using the A-CHESS recovery support tool to assess active and passive relapse risks and predict lapses to alcohol use with good sensitivity and specificity.
- The revised application has incorporated greater discussion and measurement of opioid specific risk factors, including symptoms of pain, pain catastrophizing, and medication assisted treatment, which adds to the significance.

Weaknesses

- None.

2. Investigator(s):

Strengths

- MPIs, Drs. Curtin and Shah, bring requisite expertise in addiction medicine, statistical methodology, relapse risk factors, mobile technology, and health communications. The PIs are supported by an exceptional team of Co-Is that have expertise in machine learning and passive monitoring of text/voice/face data.
- The Multiple PI plan is excellent.
- The team has already been collaborating on two other R01s that are examining opioid use disorder and alcohol use disorder using the A-CHESS environment.

Weaknesses

- None.

3. Innovation:

Strengths

- Using multiple data sources that are integrated within the A-CHESS system is highly innovative and has high potential for future innovation in improving recovery support for individuals with opioid and other substance use disorders.
- The machine learning approaches are modestly innovative and have great potential to increase our understanding of static and dynamic relapse risk factors.
- Conducting analyses with less data than is available is also a highly innovative and creative aspect of the design.

Weaknesses

- None.

4. Approach:

Strengths

- The plan for recruitment of a diverse sample is a strength.
- The focus on both initial abstinence and protracted abstinence, stratified by sex, is a strength of the design that is likely to yield novel findings about the timing of lapse risk factors in the process of behavior change.
- The stream of active and passive data collection is a strength that will potentially increase dissemination potential.
- Focusing on contextual factors and social network factors, including voice and text message contexts within the social network is a strength.
- Preliminary data provides evidence that the team is successful in recruiting and retaining individuals in trials of A-CHESS.

Weaknesses

- Aim 3 remains underdeveloped in the revised application. It is unclear how or when the lapse risk prediction tool will be implemented in A-CHESS and whether the implementation will be evaluated and how it would be evaluated. The dissemination to clinical care is a valuable next step for the research assuming that the risk prediction tool is effective, however it is unclear what the process will be for implementation. This aspect of the application is not described and lacks rigor.

- The application is relatively silent on sex differences in lapse risk and analyses that incorporate sex as a biological variable, although the stratification of individuals in the different abstinence groups by sex is a strength.

5. Environment:

Strengths

- The environment at the University of Wisconsin-Madison is exceptional to support the proposed work, including expertise in addiction, mobile health, and digital communication technology.

Weaknesses

- None.

Study Timeline:

- Not applicable.

Protections for Human Subjects:

Acceptable Risks and/or Adequate Protections

- The risks, particularly with respect to privacy and confidentiality, are extensive, but the team has adequate protections in place and has prior expertise in securing mobile health data from multiple streams.

Data and Safety Monitoring Plan (Applicable for Clinical Trials Only):

Not Applicable (No Clinical Trials)

Inclusion of Women, Minorities and Children:

- Sex/Gender: Distribution justified scientifically
- Race/Ethnicity: Distribution justified scientifically
- For NIH-Defined Phase III trials, Plans for valid design and analysis: Not applicable
- Inclusion/Exclusion of Children under 18: Excluding ages <18; justified scientifically
- Approximately equal numbers of males and females will be recruited. Ethnic and racial minorities will be oversampled to increase representation. Children will be excluded, which is justified.

Vertebrate Animals:

Not Applicable (No Vertebrate Animals)

Biohazards:

Not Applicable (No Biohazards)

Resubmission:

- The application was mostly responsive to prior critiques, including adding an expert in opioid use disorder, adding several measures to assess pain, physical health, quality of life, and medication assisted treatment. The applicants also improved the analytic plan.

- The Aim #3 implementation plan remains underdeveloped, which was a problem raised in the prior review.

Budget and Period of Support:

Recommend as Requested

CRITIQUE 2

Significance: 2
Investigator(s): 1
Innovation: 1
Approach: 3
Environment: 1

Overall Impact: This 5-year R01 proposes to apply novel machine learning methods to develop prediction models to estimate lapse likelihood in participants with opioid disorder. The team's long-term aim is to develop models capable of estimating lapse likelihood on a dynamic (i.e., day-to-day) basis, while relying largely on passive data collection methods. Using a prospective design and remote data collection methods, the team will acquire a large dataset from a cohort of abstinence-seeking participants (N=480). Data elements will consist of information collected passively /continuously via smartphone (e.g., GPS locations, text/phone logs) as well as self-report methods (e.g., daily ecological momentary assessment surveys, voice analysis of daily video check-ins). The team will then apply several machine learning techniques to develop and validate probabilistic lapse prediction models. After validating models for opioid lapses (Aim 1), Aim 2 seeks to apply the same approach to test lapse prediction models for alcohol and stimulants, while Aim 3 seeks to program the final prediction models into the team's previously validated smartphone recovery support application (A-CHESS). Overall, this is an interesting and highly innovative application that addresses a topic of urgent public health importance. The multidisciplinary research team is excellent, and the team is uniquely positioned to carry out the project (having produced the A-CHESS app). The revised proposal is responsive to previous critiques, including the important addition of a Co-Investigator with addiction medicine expertise. Detracting somewhat from the proposal are a few minor to moderate concerns in the areas of significance (e.g., uncertainty about the evidence base to support feasibility of similar dynamic prediction models) and approach (e.g., feasibility of engaging/retaining geographically remote patients in a daily assessment study for 12 months). In addition, compelling arguments as to the importance of Aims 2 and 3 in the context of this project - especially given numerous important questions that might be addressed with this highly unique dataset - seemed lacking. Nonetheless, factors weighing in favor of this proposal include its high degree of innovation, the excellent research team and environment, and the high significance of developing novel clinical support systems to address an urgent public health issue.

1. Significance:

Strengths

- The application addresses an urgent public health problem and proposes an innovative clinical application (dynamic opioid lapse prediction models based on remote data collection). If successful, this aim could have real implications from a clinical and health services perspective. Significance is very high from this standpoint.

- The aim of developing algorithms that account for interplay between stable background factors and temporally dynamic risk factors aligns strongly with theoretical models of relapse and behavior change, supporting scientific premise.
- Prior studies by this group provide initial support for the efficacy of the A-CHESS app, one of the first mHealth applications for addiction treatment. The current project could ultimately extend the clinical utility and functionality of the app in an important way.

Weaknesses

- Evidence that similar methods have produced probabilistic models that predict day-to-day dynamic behaviors with reasonably accuracy was not as clear, making for a more tentative premise in this respect. If similar approaches have been successful for other health behaviors, further discussion would strengthen the premise. Pilot results from an alcohol project are noted, but with limited detail, and seemingly involving on one data element/predictor per model.
- The arguments for Aims 2-3 are less convincing relative to Aim 1. For a project focused on opioid treatment/relapse, Aim 2 (to replicate Aim 1 in developing lapse prediction models for other drugs) seems of secondary interest – in particular in contrast to a focus on other outcomes critical for this population (e.g., medication adherence, clinic attendance, treatment re-engagement following lapses). Even absent the machine-learning aims, the project would produce an immense and unique dataset with which numerous questions could be addressed.
- The aim of adding the final risk prediction models to the A-CHESS app at the end of the project (Aim 3) was not discussed in detail, and implications of this aim (in the context of the proposed data collection) were somewhat ambiguous.

2. Investigator(s):

Strengths

- This is a high-quality team with outstanding expertise overall. The contact PI (Curtin) is a clinical psychologist with extensive expertise in substance use disorders, also possessing advanced statistical expertise. MPI Shah, the Scientific Director of CHESS, brings strong expertise in information and communication technologies and computational methods.
- MPI plan is well justified in the context of the proposal.
- The addition of Dr. Brown, an expert in addiction medicine, is an important modification that benefits the proposal.

Weaknesses

- None noted.

3. Innovation:

Strengths

- Strong innovation is evident in several respects. The proposal for large-scale, remote data collection from a high-risk clinical population over an extended period is ambitious and innovative.
- The focus on developing probabilistic models to generate dynamic estimates of next-day lapse liability is a notable and significant extension of most work in this area. The application of machine learning methods for this purpose, particularly in a naturalistic and representative sample, clearly breaks new ground.

- The project involves innovative methods of recruiting and engaging participants (e.g., video-based interviews/orientation sessions, collection of daily video check-ins, issuing payments to participants' cellular providers as a means of compensation).

Weaknesses

- None noted.

4. Approach:

Strengths

- The aim to develop algorithms based largely on low-burden, passively derived data is advantageous for eventual clinical implementation.
- Recruiting from treatment centers across the local region is a strength.
- Overall the approach benefits from the integration of diverse methods from several disciplines.
- The team is currently implementing data collection methods similar to these in ongoing projects, increasing confidence in feasibility.

Weaknesses

- The feasibility of engaging/retaining these high-risk participants on a nation-wide basis (relying on recruitment via Facebook) is unclear. Focusing on local area/clinics, where patients can be recruited/engaged directly by staff, would yield better retention rates and data quality, which may be critical. The applicants suggest that the recruitment targets could easily be met locally, which is perhaps a more pragmatic starting point.
- Although concerns about EMA compliance are often exaggerated, one year is a very long time to expect reasonable compliance with daily questionnaires and video updates.
- The A-CHESS app is, by design, intended to influence patients' behavior (e.g., notifying of high-risk locations, providing supportive content, etc.). Whether or how the app's clinical features might alter naturalistic lapse processes, and thus the final predictive models, was not addressed. This aspect of the design could impact generalizability to a greater extent than other factors emphasized in the application (e.g., geographic location).
- The machine learning aims are partly contingent on the quality of data provided by patients at the front end (e.g., addresses and phone numbers for friends, addresses of high/low risk locations, high-risk dates, etc.). From a practical standpoint, requiring the participant to input all of this info manually into the app seems arduous, which could compromise the quality/completeness of these data.
- If there is a downside to the sole focus on machine learning aims, it's greater uncertainty about the likelihood of success (which impacts all project aims). This does not reduce the quality of the approach, but makes for a high-risk/high-reward project.

5. Environment:

Strengths

- The environment at Wisconsin, including the Investigators' laboratories, appears excellent.
- The CHESS center is, by definition, the ideal site to support this project.
- The team has demonstrated collaborations with local treatment sites.

Weaknesses

- None noted.

Study Timeline:

- Not applicable.

Protections for Human Subjects:

Acceptable Risks and/or Adequate Protections

- The comprehensive data collection plan raises numerous privacy considerations, but it appears the team has the adequate protections in place. For instance, patients can opt out of providing certain types of information.

Data and Safety Monitoring Plan (Applicable for Clinical Trials Only):

Inclusion of Women, Minorities and Children:

- Sex/Gender: Distribution justified scientifically
- Race/Ethnicity: Distribution justified scientifically
- For NIH-Defined Phase III trials, Plans for valid design and analysis: Not applicable
- Inclusion/Exclusion of Children under 18: Including ages <18; not justified scientifically
- The team will make active efforts to recruit racial/ethnic minority groups to prevent under-representation. Recruitment efforts will also ensure a balanced sex distribution.

Vertebrate Animals:

Not Applicable (No Vertebrate Animals)

Biohazards:

Not Applicable (No Biohazards)

Resubmission:

- The application is responsive to prior comments, and includes several additions that strengthen the project. A key change is the addition of Dr. Brown, an addiction medicine specialist with extensive expertise. Additional pilot results and recruitment estimates are provided to support feasibility. Several assessment approaches are clarified (e.g., pain is now measured daily), and the application provides updated methods for machine learning analyses.

Budget and Period of Support:

Recommend as Requested

CRITIQUE 3

Significance: 1
Investigator(s): 1
Innovation: 1
Approach: 2
Environment: 1

Overall Impact: This application proposes a truly innovative, highly significant study that would be carried out by an exceptional collaborative multi-disciplinary team. A lapse prediction model would be developed using machine learning methods on the basis of distal, static risk signals and proximal, time-varying risk signals and would be integrated into real-time clinical care using the Comprehensive Health Enhancement Support System for Addiction (A-CHESS) mobile app. This would provide “just-in-time” support to the patient and could efficiently alert a clinician during a time of high lapse potential. The project is novel in its collection of multiple streams of active and passive data through Smartphone sensors, natural language processing, etc., and importantly, this information would be contextualized to people, places, and situations that support or undermine an individual’s abstinence efforts. Collection of such a wide array data sources over a 12-month period in a large sample (N=480) of a difficult population will be challenging, although preliminary data and the team’s expertise assuage concerns a bit. Regardless, enthusiasm for this exciting and important study is very high.

1. Significance:

Strengths

- Opioid use disorder treatment outcomes are poor due to high relapse rates. Understanding the factors that precipitate lapse is critical, but most research to date focuses on distal, static characteristics of the person and fails to consider the proximal risk factors. Establishing a means of identifying real-time changes in risk would advance the field greatly.
- Recent research has begun developing lapse risk prediction models using machine learning. Integrating these risk prediction models into real-time clinical care would provide support to the patient when needed; moreover, real-time information can be delivered to clinicians about their patient’s risks and information can be integrated into the electronic health record.
- Opioid lapse risk prediction models will be extended to alcohol or other substances. This permits understanding of whether the best risk prediction signals are generic or substance-specific and is also important because individuals with opioid use disorder frequently have comorbid substance use disorders.

Weaknesses

- None noted.

2. Investigator(s):

Strengths

- MPI Curtin is a well-established and productive researcher with a history of NIH funding who has expertise in the etiology of addiction and longitudinal research design as well as real-world assessment via mobile technology. MPI Shah is a leading communication scientist with expertise in the use of computational methods such as natural language processing and computational linguistics.
- The Multiple PI Leadership Plan describes the roles and responsibilities for each PI; if consensus cannot be reached among the MPIs, input will be solicited from the entire investigative team.
- The multi-disciplinary, complementary, highly collaborative research team is stellar. The group includes both the Scientific Director and Director of CHESS, the developer of the original A-CHESS platform, and an expert in the treatment of opioid use disorder (Co-I Brown) who was newly added to the research team; he will also facilitate participant recruitment through the local community.

- The investigators currently have an NIAAA funded project to conduct a similar (but smaller scope) study on alcohol lapse risk prediction that will inform the proposed study; the research team is also conducting a study with A-CHESS with adjunct to medication assisted treatment for opioid use disorder. This experience with these similar projects support the proposed project's feasibility and likelihood of success.

Weaknesses

- None noted.

3. Innovation:

Strengths

- Truly innovative in its collection of lapse risk signals via multiple data sources, including daily and bi-monthly self-report; GPS tracking; linguistic analysis of text message content and video check-ins (as well as extraction of acoustic and image data); text and voice call logs.
- Temporally proximal risk signals have accurate predictive utility and may potentially serve as targets for mHealth just-in-time intervention content.
- By contextualizing the signals, it will be possible to pinpoint in real-time the participant's exposure to high-risk people, places, and situations.
- Use of innovative machine learning modeling model development, training, and validation.
- Integration of real-time lapse prediction into an existing mHealth platform.

Weaknesses

- None noted.

4. Approach:

Strengths

- A large number of participants with opioid use disorder (N=480) who are pursuing abstinence will be followed for 12 months. Relapse signals will be assessed at the distal and proximal level through active and passive Smartphone-based data collection. The design is rigorous, thoughtful, and based on the investigators' prior work.
- To contextualize risk signals, participants will provide information about people and places that they consider to support or undermine their abstinence efforts. This content will be integrated with passive and active data, along with objective information about low-risk contexts (e.g., treatment center) and high-risk contexts (e.g., very low activity level).
- Video recruitment/training will be done when recruitment and training cannot be conducted in person.
- Enrolling participants across a range of periods of abstinence allows for detection of lapse risk signals that span early vs. later periods of recovery.
- A-CHESS is shown to be successfully used by a large number of research participants and patients. It proves to be user friendly and is optimized to collect data with minimal impact on battery life.
- Preliminary data supporting feasibility and effectiveness of using simpler real-time risk prediction models with fewer features using similar machine learning methods are given.
- One of the study goals is to compare full models with all risk signals with simpler models that ignore specific signals in order to determine whether the more sparse sets perform equally well,

thereby reducing burden. Comparing survey-level data to data aggregated across surveys will also be informative for determining sampling frequency.

- Will test differences by sex (key biological variable) given evidence that some signals may be more lapse-inducing for women than men.

Weaknesses

- Recruitment, retention, and data collection with such a large sample over such a long period of time will be challenging. Preliminary data and the team's expertise assuage concerns a bit but do not lessen the magnitude of what the investigators are trying to do.

5. Environment:

Strengths

- The Addiction Research Center (ARC) at the University of Wisconsin-Madison will provide the necessary resources for carrying out the project.
- The Center for Health Enhancement Systems Studies (CHESS) uses technology such as mHealth apps deployed via smartphones to improve health and access to care. It is a highly interdisciplinary center, with each of the investigators on the project highly involved in CHESS projects.
- The Center for High Throughput Computing (CHTC) offers substantial support for the project's demanding computational needs (machine learning model training, cross validation).

Weaknesses

- None noted.

Study Timeline:

Protections for Human Subjects:

Acceptable Risks and/or Adequate Protections

- Adequate protections are in place for the primary risks of breach of confidentiality, use of a Smartphone for data collection, and report of third-party information. Data are collected and stored on the study secure server and are never saved locally on the phone.
- Means of ensuring privacy (for both participant and third-party) through opt-out of a given data source is now described, although it is also noted that the investigators' current research with similar populations obtains high rates of compliance with similar assessments.

Data and Safety Monitoring Plan (Applicable for Clinical Trials Only):

Not Applicable (No Clinical Trials)

Inclusion of Women, Minorities and Children:

- Sex/Gender: Distribution justified scientifically
- Race/Ethnicity: Distribution justified scientifically
- For NIH-Defined Phase III trials, Plans for valid design and analysis: Not applicable
- Inclusion/Exclusion of Children under 18: Excluding ages <18; justified scientifically
- Equal numbers of men and women will be recruited. Racial/minority groups will be over-sampled, with the final sample anticipated to be 30% non-White; 20% Hispanic.

- The focal population is adults age 18 years and older.

Vertebrate Animals:
Not Applicable (No Vertebrate Animals)

Biohazards:
Not Applicable (No Biohazards)

Resubmission:
- The investigators have been highly responsive to prior reviewer comments.
- It is clarified that the A-CHESS Recovery support app is the platform through which all active and passive real-time data are being collected. Using this app also improves feasibility of implementation when the lapse prediction models are added to A-CHESS (Aim 3).
- Local recruitment will now supplement national recruitment and will also serve to initially pilot procedures and materials.
- Several opioid-specific measures related to treatment engagement and lapse have been added to the surveys and will be included in the models. Chronic pain, pain catastrophizing, general health functioning, and quality of life have been added. Self-report and passive assessment of medication assisted treatment (MAT) use/adherence is also clarified as well as EMA assessment of opioid craving and opioid abstinence self-efficacy.
- Clarifications about the psychometrics of self-report measures and diagnosis with opioid use disorder were made.
- More information is provided with regard to extraction of facial features from video check-ins by Co-I Sethares as well as through FaceReader algorithms.
- Improvements have been made to the machine learning algorithms and cross-validation models. Detail about data pre-processing has been added.

Budget and Period of Support:
Recommend as Requested

THE FOLLOWING SECTIONS WERE PREPARED BY THE SCIENTIFIC REVIEW OFFICER TO SUMMARIZE THE OUTCOME OF DISCUSSIONS OF THE REVIEW COMMITTEE, OR REVIEWERS' WRITTEN CRITIQUES, ON THE FOLLOWING ISSUES:

PROTECTION OF HUMAN SUBJECTS: ACCEPTABLE

INCLUSION OF WOMEN PLAN: ACCEPTABLE

INCLUSION OF MINORITIES PLAN: ACCEPTABLE

INCLUSION OF CHILDREN PLAN: ACCEPTABLE

COMMITTEE BUDGET RECOMMENDATIONS: The budget was recommended as requested.

Footnotes for 1 R01 DA047315-01A1; PI Name: Curtin, John J.

NIH has modified its policy regarding the receipt of resubmissions (amended applications). See Guide Notice NOT-OD-14-074 at http://grants.nih.gov/grants/guide/notice-files/NOT-OD-14-074.html. The impact/priority score is calculated after discussion of an application by averaging the overall scores (1-9) given by all voting reviewers on the committee and multiplying by 10. The criterion scores are submitted prior to the meeting by the individual reviewers assigned to an application, and are not discussed specifically at the review meeting or calculated into the overall impact score. Some applications also receive a percentile ranking. For details on the review process, see http://grants.nih.gov/grants/peer_review_process.htm#scoring.

MEETING ROSTER

Addiction Risks and Mechanisms Study Section
Risk, Prevention and Health Behavior Integrated Review Group
CENTER FOR SCIENTIFIC REVIEW
ARM
02/11/2019 - 02/12/2019

Notice of NIH Policy to All Applicants: Meeting rosters are provided for information purposes only. Applicant investigators and institutional officials must not communicate directly with study section members about an application before or after the review. Failure to observe this policy will create a serious breach of integrity in the peer review process, and may lead to actions outlined in NOT-OD-14-073 at https://grants.nih.gov/grants/guide/notice-files/NOT-OD-14-073.html and NOT-OD-15-106 at https://grants.nih.gov/grants/guide/notice-files/NOT-OD-15-106.html, including removal of the application from immediate review.

CHAIRPERSON(S)

WITKIEWITZ, KATIE, PHD
REGENTS PROFESSOR
DEPARTMENT OF PSYCHOLOGY
UNIVERSITY OF NEW MEXICO
ALBUQUERQUE, NM 87131

MEMBERS

CHAPLIN, TARA M, PHD *
ASSOCIATE PROFESSOR
DEPARTMENT OF PSYCHOLOGY
GEORGE MASON UNIVERSITY
FAIRFAX, VA 22030

CORBIN, WILLIAM R, PHD
PROFESSOR
DEPARTMENT OF PSYCHOLOGY
ARIZONA STATE UNIVERSITY
TEMPE, AZ 85287

DAUGHTERS, STACEY B, PHD
PROFESSOR
DEPARTMENT OF PSYCHOLOGY AND NEUROSCIENCE
UNIVERSITY OF NORTH CAROLINA, CHAPEL HILL
CHAPEL HILL, NC 27599

FOX, HELEN C, PHD *
ASSISTANT PROFESSOR
DEPARTMENT OF PSYCHIATRY
STONY BROOK UNIVERSITY SCHOOL OF MEDICINE
STONY BROOK, NY 11794

HASIN, DEBORAH S, PHD *
PROFESSOR
DEPARTMENT OF PSYCHIATRY
MAILMAN SCHOOL OF PUBLIC HEALTH
AND COLLEGE OF PHYSICIANS AND SURGEONS
COLUMBIA UNIVERSITY
NEW YORK, NY 10032

HEITZEG, MARY M, PHD
ASSOCIATE PROFESSOR
DEPARTMENT OF PSYCHIATRY
AND ADDICTION CENTER
UNIVERSITY OF MICHIGAN
ANN ARBOR, MI 48109

HENDERSHOT, CHRISTIAN S, PHD *
ASSOCIATE PROFESSOR
DEPARTMENT OF PSYCHIATRY
UNIVERSITY OF TORONTO
TORONTO, ON M6J 1H4

HOPFER, CHRISTIAN J, MD
PROFESSOR
DEPARTMENT OF PSYCHIATRY
SCHOOL OF MEDICINE
UNIVERSITY OF COLORADO AT DENVER
AURORA, CO 80045

JACKSON, KRISTINA M, PHD
RESEARCH PROFESSOR
DEPARTMENT OF BEHAVIORAL AND SOCIAL SCIENCES
CENTER FOR ALCOHOL AND ADDICTION STUDIES
BROWN UNIVERSITY
PROVIDENCE, RI 02912

JOHNSON, MATTHEW W, PHD
ASSOCIATE PROFESSOR
DEPARTMENT OF PSYCHIATRY
AND BEHAVIORAL SCIENCES
BEHAVIORAL PHARMACOLOGY RESEARCH UNIT
JOHNS HOPKINS UNIVERSITY SCHOOL OF MEDICINE
BALTIMORE, MD 21224

KERR, WILLIAM C, PHD
SENIOR SCIENTIST
ALCOHOL RESEARCH GROUP
PUBLIC HEALTH INSTITUTE
EMERYVILLE, CA 94608

LEVE, LESLIE D, PHD
PROFESSOR
ASSOCIATE VP FOR RESEARCH
COLLEGE OF EDUCATION
UNIVERSITY OF OREGON
EUGENE, OR 97403

LISDAHL, KRISTA M, PHD
ASSOCIATE PROFESSOR
DEPARTMENT OF PSYCHOLOGY
UNIVERSITY OF WISCONSIN-MILWAUKEE
MILWAUKEE, WI 53221

LUCIANA, MONICA, PHD
PROFESSOR
DEPARTMENT OF PSYCHOLOGY
UNIVERSITY OF MINNESOTA
MINNEAPOLIS, MN 55455

LUNDAHL, LESLIE H, PHD *
ASSOCIATE PROFESSOR
SUBSTANCE ABUSE RESEARCH DIVISION
DEPARTMENT OF PSYCHIATRY AND
BEHAVIORAL NEUROSCIENCES
WAYNE STATE UNIVERSITY SCHOOL OF MEDICINE
DETROIT, MI 48201

MARTIN, LAURA E, PHD *
ASSOCIATE PROFESSOR
DEPARTMENT OF PREVENTIVE MEDICINE
AND PUBLIC HEALTH, HOGLUND BRAIN IMAGING CENTER
UNIVERSITY OF KANSAS MEDICAL CENTER
KANSAS CITY, KS 66160

MCCARTHY, DANIELLE E, PHD *
ASSOCIATE PROFESSOR
DEPARTMENT OF MEDICINE
CENTER FOR TOBACCO RESEARCH AND INTERVENTION
UNIVERSITY OF WISCONSIN SCHOOL OF MEDICINE
MADISON, WI 53711

MUN, EUN-YOUNG, PHD
PROFESSOR
DEPARTMENT OF HEALTH BEHAVIOR
AND HEALTH SYSTEMS
SCHOOL OF PUBLIC HEALTH
UNIVERSITY OF NORTH TEXAS HEALTH SCIENCE CENTER
FORT WORTH, TX 76107

NAGEL, BONNIE J, PHD
PROFESSOR
DEPARTMENTS OF PSYCHIATRY
AND BEHAVIORAL NEUROSCIENCE
OREGON HEALTH AND SCIENCE UNIVERSITY
PORTLAND, OR 97239

POKHREL, PALLAV, PHD *
ASSOCIATE PROFESSOR
POPULATION SCIENCES IN THE PACIFIC PROGRAM
CANCER PREVENTION IN THE PACIFIC
UNIVERSITY OF HAWAII CANCER CENTER
HONOLULU, HI 96813

POTTER, JENNIFER S, PHD *
VICE DEAN FOR RESEARCH, PROFESSOR OF PSYCHIATRY
LONG SCHOOL OF MEDICINE
UNIVERSITY OF TEXAS HEALTH SCIENCE CENTER
SAN ANTONIO
SAN ANTONIO, TX 78229

SAMEK, DIANA R, PHD *
ASSOCIATE PROFESSOR
DEPARTMENT OF HUMAN DEVELOPMENT
& FAMILY STUDIES
AUBURN UNIVERSITY
AUBURN, AL 36849

SCHERRER, JEFFREY F, PHD *
PROFESSOR
DEPARTMENT OF FAMILY AND COMMUNITY MEDICINE
RESEARCH DIVISION
ST. LOUIS UNIVERSITY SCHOOL OF MEDICINE
ST. LOUIS, MO 63104

SIMMONS, VANI N, PHD
ASSOCIATE MEMBER
DEPARTMENT OF HEALTH
OUTCOMES AND BEHAVIOR
MOFFITT CANCER CENTER AND RESEARCH INSTITUTE
TAMPA, FL 33647

SIMONS, JEFFREY S, PHD
PROFESSOR
DEPARTMENT OF PSYCHOLOGY
UNIVERSITY OF SOUTH DAKOTA
VERMILLION, SD 57069

STERLING, KYMBERLE L, PHD
ASSOCIATE PROFESSOR
HEALTH PROMOTION AND BEHAVIORAL SCIENCES
UNIVERSITY OF TEXAS HEALTH SCIENCES CENTER
SCHOOL OF PUBLIC HEALTH, DALLAS CAMPUS
DALLAS, TX 75390

STRASSER, ANDREW A, PHD
ASSOCIATE PROFESSOR
DEPARTMENT OF PSYCHIATRY
ABRAMSON CANCER CENTER
PERELMAN SCHOOL OF MEDICINE
THE UNIVERSITY OF PENNSYLVANIA
PHILADELPHIA, PA 19104

SUTHERLAND, MATTHEW T, PHD *
ASSISTANT PROFESSOR
DEPARTMENT OF PSYCHOLOGY
FLORIDA INTERNATIONAL UNIVERSITY
MIAMI, FL 33199

SCIENTIFIC REVIEW OFFICER

PRENTICE, KRISTEN, PHD
SCIENTIFIC REVIEW OFFICER
CENTER FOR SCIENTIFIC REVIEW
NATIONAL INSTITUTES OF HEALTH
BETHESDA, MD 20892

EXTRAMURAL SUPPORT ASSISTANT

WATTS, MELISSA D
EXTRAMURAL SUPPORT ASSISTANT
CENTER FOR SCIENTIFIC REVIEW
NATIONAL INSTITUTE FOR HEALTH
BETHESDA, MD 20892

* Temporary Member. For grant applications, temporary members may participate in the entire meeting or may review only selected applications as needed.

Consultants are required to absent themselves from the room during the review of any application if their presence would constitute or appear to constitute a conflict of interest.
